# New software

**DOI:** 10.1155/S1463924602000044

**Published:** 2002


					New software
Alias Limited release I-View
Alias Limited, market leader for piping isometric soft-
ware, today announced the availability of I-View, a 3D
pipe visualization tool. I-View is capable of handling
single or multiple pipes from any 3D plant design system
supporting ISOGEN. Even piping (R) les from dia erent
systems can be viewed as a single model. This new
module now provides I-Sketch users with the ability to
view pipes in true scale and to clash-check the piping
systems visually.
The I-View model can be rotated to any angle, or the
user can select a default view from a drop-down list.
Individual pipe attributes are displayed by simply select-
ing a pipe; information, such as the speci(R) cation, pipe
length and bore, will be shown in the information panel.
I-View has been developed to use the familiar Windows
interface and will be available as a plug-in to Internet
Explorer to enable collaborative work processes via an
Internet or Intranet network.
I-View is of great bene(R) t to all companies involved in the
production of piping isometrics.
For more information about I-View please visit the Alias web site
www.alias.ltd.uk, or telephone or e-mail the Commercial Man-
ager, Andy Osborne, on Tel: + 44(0)1928 579311, e-mail:
andy.osborne@alias.ltd.uk
Journal of Automated Methods & Management in Chemistry
Vol. 24, No. 1 (January-February 2002) p. 25
Journal of Automated Methods & Management in Chemistry ISSN 1463 9246 print/ISSN 1464 5068 online # 2002 Taylor & Francis Ltd
http://www.tandf.co.uk/journals
DOI: 10.1080/14639240210132345
25

				

